# Healthcare expenditure and technology use in pediatric diabetes care

**DOI:** 10.1186/s12902-023-01316-3

**Published:** 2023-04-07

**Authors:** Silvia A. G. de Vries, Jessica C. G. Bak, Carianne L. Verheugt, Vincent A. Stangenberger, Dick Mul, Michel W. J. M. Wouters, Max Nieuwdorp, Theo C. J. Sas

**Affiliations:** 1 Department of Vascular Medicine, Amsterdam University Medical Centers, Amsterdam, The Netherlands; 2 Dutch Institute for Clinical Auditing, Leiden, The Netherlands; 3 Hospital & Health Care, LOGEX, Amsterdam, The Netherlands; 4 Diabeter, Center for Pediatric and Adult Diabetes Care and Research, Rotterdam, The Netherlands; 5 Department of Biomedical Data Sciences, Leiden University Medical Center, Leiden, The Netherlands; 6 Department of Pediatrics, Division of Pediatric Endocrinology, Erasmus University Medical Center, Sophia Children’s Hospital, Rotterdam, The Netherlands

**Keywords:** Children, Diabetes mellitus, Healthcare utilization, Hospital costs, Technology, Continuous glucose monitor

## Abstract

**Background:**

Diabetes mellitus is one of the most common chronic diseases in childhood. With more advanced care options including ever-evolving technology, allocation of resources becomes increasingly important to guarantee equal care for all. Therefore, we investigated healthcare resource utilization, hospital costs, and its determinants in Dutch children with diabetes.

**Methods:**

We conducted a retrospective, observational analysis with hospital claims data of 5,474 children with diabetes mellitus treated in 64 hospitals across the Netherlands between 2019–2020.

**Results:**

Total hospital costs were €33,002,652 per year, and most of these costs were diabetes-associated (€28,151,381; 85.3%). Mean annual diabetes costs were €5,143 per child, and treatment-related costs determined 61.8%. Diabetes technology significantly increased yearly diabetes costs compared to no technology: insulin pumps € 4,759 (28.7% of children), Real-Time Continuous Glucose Monitoring € 7,259 (2.1% of children), and the combination of these treatment modalities € 9,579 (27.3% of children). Technology use increased treatment costs significantly (5.9 – 15.3 times), but lower all-cause hospitalisation rates were observed. In all age groups, diabetes technology use influenced healthcare consumption, yet in adolescence usage decreased and consumption patterns changed.

**Conclusions:**

These findings suggest that contemporary hospital costs of children with diabetes of all ages are driven primarily by the treatment of diabetes, with technology use as an important additive factor. The expected rise in technology use in the near future underlines the importance of insight into resource use and cost-effectiveness studies to evaluate if improved outcomes balance out these short-term costs of modern technology.

**Supplementary Information:**

The online version contains supplementary material available at 10.1186/s12902-023-01316-3.

## Background

Diabetes mellitus is one of the most common chronic childhood diseases and the incidence rates will continuously rise [1]. International predictions illustrate an increase in patients with diabetes in the following decades, resulting in a lifelong diabetes-associated burden for millions of children worldwide [2, 3]. In the Netherlands, the number of patients with diabetes is expected to rise annually until at least 2040 as well [4]. In 2019, approximately 10,000 children in the Netherlands had a diagnosis of diabetes, and the incidence rate of type 1 diabetes has previously been estimated between 21–30 per 100,000 children [4, 5].

As long as a curative treatment has yet to be discovered, optimal treatment of diabetes and prevention of serious long-term complications remain chronic necessities. Evidence has repeatedly pointed out high complication risks, increased mortality rates, and negative impact on quality of life in patients with youth-onset diabetes [6, 7]. Initiatives to improve patient care have further evolved to turn this tide. A patient-centered approach, multidisciplinary care, and technologic advances are increasingly finding their place in modern diabetes practice. New treatment modalities such as flash glucose monitoring, continuous glucose monitoring, and advances in (closed-loop) continuous subcutaneous insulin infusion have shown promising results in patient outcomes and diabetes burden [8].

With growing patient numbers and advancing care options, optimal allocation of healthcare resources becomes increasingly important. This is illustrated by a recent study showing an increase in costs partially driven by the utilization of new diabetes technology [9]. Beneficial effects on outcomes may well balance out these additional costs in the long run, but insight in healthcare utilization and costs is greatly warranted to equally and efficiently distribute available resources. The few studies that investigated healthcare use and costs in pediatric diabetes care were not on a national level or not recently performed, thus not reflecting current pediatric diabetes practices [10–19]. Previously reported factors to influence costs were medication, hospitalisations, and the use of technology. Some studies also observed increasing costs over the years [9, 12]. Contemporary costs are expected to rise further due to new technologic opportunities, however, current healthcare resource utilization of Dutch children with diabetes and its contributing factors are unclear.

Using nationwide healthcare reimbursement data, we sought to investigate healthcare resource utilization, hospital costs, and its determinants in Dutch children and adolescents (0–18 years of age) with diabetes mellitus.

## Methods

### Study design

We conducted a retrospective, observational, nationwide cohort study using Dutch healthcare reimbursement data. In Dutch healthcare, the reimbursement of hospital care activities is organized through a national diagnosis coding system, the Diagnosis Treatment Combinations (DBC) system [20]. All DBC care products (DBCs) are centrally registered and collected in the hospital information systems (HIS) in each hospital. These DBC claims consist of information on the diagnosis, medical specialty, and treatment activities. For this study, a dataset was obtained from a database serviced by LOGEX (Amsterdam, the Netherlands) that contains benchmark information from affiliated hospitals based on claimed care products in local HIS. Previous studies have shown that administrative databases from sources like the LOGEX database can reliably be used for quality assessment of Dutch healthcare [21, 22]. Basic health insurance is mandatory by Dutch law, and insurance for children up to 18 years old is free. All healthcare costs are covered via the reimbursement system, so it is reasonably assumed that all care activities and corresponding costs of each individual included are covered. In the Netherlands, all children diagnosed with diabetes mellitus are treated by pediatricians based in hospitals, diabetes-oriented collaborations between hospitals, or independent clinics specialized in diabetes care. In case of diabetes complications in the young, care may be provided by ophthalmologists or in rare cases by (orthopedic) surgeons. In 2019, the LOGEX benchmark database comprised 65 affiliated Dutch secondary and academic hospitals, covering approximately 88% of all hospitals in the Netherlands. One hospital did not treat any children with diabetes. Independent treatment clinics were not included (~ 22% of Dutch children with diabetes) as this data was not available. Every delivery of reimbursement data from hospitals to LOGEX is validated by comparing data with previous data deliveries and hospital electronic health record data; in case of inconsistencies hospitals are asked to evaluate or redeliver data.

### Data collection

Children aged 0 up to and including 17 years with one or more diabetes DBC claims between 1 January 2019 and 31 December 2019 were included. Included individuals had a follow-up duration of 365 consecutive days after the registration date of the claim in 2019, creating a follow-up duration of one year for each patient. All claims of the following five medical specialties were collected: Pediatrics, Internal medicine, Surgery (diagnosis codes for diabetic foot), Orthopaedics (diagnosis codes for diabetic foot), and Ophthalmology (diagnosis codes for diabetic retinopathy and maculopathy). Specialty codes and diagnosis codes can be found in supplementary table S1. Patients were excluded if the date of birth was missing (*n* = 3). All data used for this study were de-identified, rendering data untraceable to individual patients in the data analysis. Therefore, no ethical approval or informed consent was required. Random identification numbers were assigned to each patient to allow for follow-up over time in the dataset. Sex, age categories, socio-economic status (SES), hospital of treatment, and survival status of each patient were collected. SES was determined by The Netherlands Institute for Social Research, based on average income, percentage of unemployed individuals, and percentage of less educated individuals, and was assessed by living area deduced by zip code [23]. SES score was graded from 0–3 for all patients, classified as respectively unknown, high, intermediate, or low. Hospitals were randomly coded from 0 to 64 to guarantee anonymity. Age was provided by categories of 5 years (0; 1–5; 6–10; 11–15; 16–17). Costs were available for all individual patients. Average costs per hospital care activity in all Dutch hospitals were calculated by LOGEX using an activity-based costing method (top-down costing) according to the costing manual of Dutch health economic guidelines [24]. Total costs were estimated by multiplying the number of hospital care activities in diabetes patients received by the value of each hospital care activity. Diabetes-specific costs were calculated similarly, except only healthcare activities performed within a reimbursed diabetes care trajectory were included. The data on reimbursed healthcare expenditure was not corrected for Consumer Price Index (CPI) because, in the Netherlands, insured healthcare costs are not considered in the calculation of the CPI [25].

### Outcomes

Primary outcome was the annual healthcare resource utilization (HCRU) of Dutch children treated for diabetes mellitus in 2019 during one year of follow-up. HCRU was expressed as the number of hospitalisations, consultations (comprising face-to-face, telephone, and e-consultations) in different specialties and healthcare activities related to the usage of insulin pumps and real-time Continuous Glucose Monitoring (rtCGM) for each patient. Healthcare activities are activities performed by the treating medical team of a patient, registered for reimbursement purposes. Technology use was defined as at least one care activity related to the use of rtCGM, an insulin pump, or the use of both treatment modalities (for activity codes see supplementary table S2). Intermittently scanned CGM was not included, because it was not yet registered separately by the Dutch reimbursement system. The secondary outcome measures were the total healthcare costs of Dutch children with diabetes and the diabetes-specific costs during the follow-up period. The evaluation of costs was conducted from a hospital perspective because the analysis included in-hospital costs only. All claimed outpatient and in-hospital expenses in the clinical care of diabetes patients were represented in the total healthcare costs, including costs unrelated to diabetes care. Diabetes-specific costs were defined as activities performed within a reimbursed diabetes care trajectory. Both cost outcomes were divided into five subcategories according to resource use: clinical costs (all costs related to hospital admissions), diagnostic costs (all diagnostic activities such as imaging or laboratory activities), additional costs (containing add-on medicine defined as medication exceeding expenses of 10,000 euro per patient per year and traveling costs), treatment costs (all treatments, i.e. surgery, minor interventions, day care treatment, rtCGM devices, activities related to technology use, supporting activities including blood products and paramedical care, but does not include medication or consumables like test strips, pens, and needles) and consultation costs (consultations by physicians and diabetes care nurses, emergency department consultations, intercollegiate consultations, or multidisciplinary consultation meetings).

### Statistical analyses

Descriptive statistics were used to assess baseline characteristics. For continuous variables with normal distribution of data, means with standard deviation (SD) were given, for all other continuous variables median with range was stated. For categorical variables, proportions were used. Hospitalisation rate was determined per 100 person-years with the total number of hospitalisations divided by the years of follow-up of all patients, multiplied by 100. Despite right-skewed distributions, all cost outcomes were expressed as mean cost per patient in Euro (€) because average costs have been described as the most informative measure for comparison of cost outcomes [26].Due to the skewness of data, we also present median costs. Interquartile ranges were used to describe the distribution of the costs [27]. All expenditures are reported in euros (1 euro = 1.04 US dollars– according to the exchange rate of 10–13–2022). Total costs were calculated by the sum of the expenses for all patients and stratified per service category (consultation costs, clinical costs, treatment costs, diagnostic costs, and additional costs). Costs and cost categories were stratified by age categories and by technology use. Because of skewness of costs data, differences between groups were tested for significance with Mann–Whitney U and Kruskal–Wallis tests, Dunn’s test was used for pairwise comparison between no technology users and technology categories. A significance level of 0.05 was used for all tests. Statistical analyses were performed using R Statistical Software (v4.0.3; R Core Team 2020).

## Results

### Baseline characteristics

Table 1 shows the characteristics of patients with diabetes mellitus (DM) treated in 64 hospitals across the Netherlands during the year 2019. A total of 5,474 children < 18 years old were included in the study population with a follow-up of one year. Sex was male in 52.0%, mean age was 12.3 ± 3.7 years, and 95.0% of patients were treated in the pediatric department. Socio-economic status (SES) was high in 34.9%, intermediate in 31.7%, low in 33.1% of patients, and unknown in 0.4% of patients. The number of children treated per hospital varied from 4 to 406. Of all patients, 20.0% visited the ophthalmology department for a diabetes-related reason, including screening. 873 patients were treated by both a pediatrician and an ophthalmologist and 52.2% of these children were between 11–15 years old. 60 children (1.1%) were treated at the internal medicine department with a diabetes diagnosis, and 217 children (4.0%) were only treated outside the pediatric or internal medicine department in the included hospitals.

**Table 1:** Baseline characteristics of the study population (*n* = 5,474)

		N	%
Sex			
	Male	2,848	52.0
	Female	2,626	48.0
Age (years)		12.3 ± 3.7	
Age group (years)			
	0	6	0.1
	1–5	336	6.1
	6–10	1,119	20.4
	11–15	2,528	46.2
	16–17	1,485	27.1
SES			
	Low	1,811	33.1
	Intermediate	1,735	31.7
	High	1,908	34.9
	Unknown	20	0.4
Mortality		1	
Hospital of treatment	Secondary	5,279	96.4
	Tertiary	195	3.6
Treating medical specialty*			
	Pediatrics	5,202	95.0
	Internal medicine	60	1.1
	Pediatrics & Internal medicine	5	0.1
	Surgery	9	0.2
	Ophthalmology	1,093	20.0
	Ophthalmology & Pediatrics	873	15.9
Diabetes-related consultations^*^			
Pediatrics	Number of consultations	7 [0—45]	
	1—3	493	9.0
	4—6	1,935	35.3
	7—9	2,118	38.7
	10—12	426	7.8
	≥ 13	213	3.9
Ophthalmology	Number of consultations	1 [1–34]	
	1	817	14.9
	2	235	4.3
	≥ 3	41	0.7
Surgery	Number of consultations	1 [0—11]	
Internal medicine	Number of consultations	2.5 [0–18]	
**All-cause hospitalisations**			
Patients with hospitalisations		1,008	18.4
	1	820	15.0
	2	133	2.4
	≥ 3	57	1.0
**Diabetes technology**			
Insulin pump care activities		3,063	56.0
	Number of care activities	7 [1—180]	
rtCGM care activities		1,608	29.4
	Number of care activities	5 [1—173]	
Insulin pump & rtCGM care activities		1,492	27.3

Numbers are presented as mean ± SD, median [range] or number of patients with percentage (%). All consultations, activities, and hospitalisations are expressed per year

*SES* Socio-economic status, *rtCGM* real-time Continuous Glucose Monitoring

^*^ Determined by a diabetes-related diagnosis treatment combination code (DBC)

### Healthcare resource utilization

Table 1 also shows the healthcare resource utilization of included patients. 5,185 patients with a diabetes-related diagnosis treatment combination code (DBC) had one or more consultations at the pediatric department. Median consultations at the pediatric department was 7 times per year (IQR 5—8). Of the 1,093 children who were treated by an ophthalmologist, 556 (50.9%) were between 11–15 years old with a median of 1 visitation. Regardless of diagnosis, 1,008 children (18.4%) were admitted to the hospital at least once, with a corresponding all-cause hospitalisation rate of 24 per 100 person-years. Among hospitalised patients, 18.8% (*n* = 190) were admitted more than once. Regarding utilization of diabetes technology, 56.0% of the study population used insulin pump therapy, and 29.4% used Real-Time Continuous Glucose Monitoring (rtCGM); 27.3% used both insulin pump and rtCGM. The median number of pump therapy care activities registered under a diabetes care trajectory was 7 per child per year. For rtCGM, the median number of annual care activities per child was 5. The hospitalisation rate for children with technology use was lower: no technology use 29 per 100 person-years, insulin pumps 20 per 100 person-years, and insulin pump & rtCGM use 19 per 100 person-years. In contrast, the group (*n* = 116) that used rtCGM only was different with a higher hospitalisation rate of 37 per 100 person-years. In this relatively small group, clinical and diagnostic costs were also markedly higher.

### Total and diabetes- associated costs

**Fig. 1:**
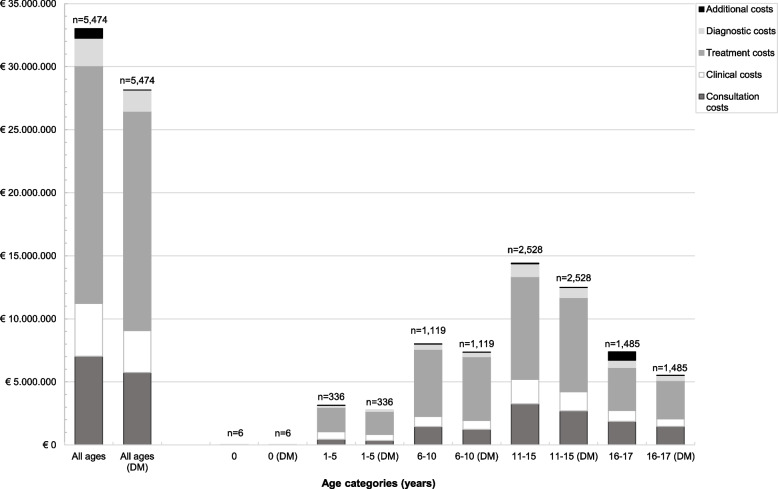
Total and diabetes-associated (DM) hospital costs for Dutch children in 2019–2020 per service category (*n* = 5,474). DM = Diabetes-associated

The total healthcare utilization costs altogether for 5,474 children were estimated at €33,002,652. Mean overall hospital annual costs were €6,029 (median €4,320, IQR 2,078 –7,644) per person. Diabetes-associated annual costs were €28,151,381 and determined most (85.3%) of the hospitals costs of these children. Mean annual diabetes-associated costs were € 5,143 (median €3,711, IQR 1,570 – 6,747) per child. The majority of diabetes-associated costs were determined by the treatment (61.8%) and consultations (20.4%) of these children, as observed in Fig. 1 and Table 2. Clinical costs, diagnostic and additional costs attributed 11.8%, 6.0%, and 0.0% respectively in children of all age groups.

**Table 2:** Total and diabetes-associated hospital costs for Dutch children of all ages in 2019–2020 per service category (*n* = 5,474)

	**Overall costs**	**Consultation costs**	**Clinical costs**	**Treatment costs**	**Diagnostic costs**	**Additional costs**
Total hospital costs all children (%)	€ 33,002,652 (100.0)	€ 7,027,891 (21.3)	€ 4,193,529 (12.7)	€ 18,852,886 (57.1)	€ 2,181,506 (6.6)	€ 746,840 (2.3)
Diabetes-associated costs all children (%)	€ 28,151,381 (100.0)	€ 5,744,939 (20.4)	€ 3,323,176 (11.8)	€ 17,400,020 (61.8)	€ 1,678,104 (6.0)	€ 5,142 (0.0)

### Treatment characteristics and diabetes-associated costs

Table 3 shows mean annual diabetes-associated costs for all children and by treatment form. Insulin pump therapy, rtCGM, and the use of both treatment modalities significantly increase the mean diabetes-associated costs when compared to no technology users (mean € 2,418, median €1,506, IQR 808—2,702). For children with care activities for an insulin pump only, this was € 4,759 (median €4,009, IQR 2,980 – 5,728), for rtCGM only € 7,259 (median €6,474, IQR 3,624 – 9,999) and in case of both this was € 9,573 (median €7,911, IQR 5,250 – 11,945). These costs are mainly caused by an exponential increase in the treatment costs category (5.9 – 15.3 times higher vs. no technology use). Consultation costs increased, and clinical costs decreased in children with technology use. Hospitalisation (≥ 1) was associated with higher mean diabetes-associated costs of €7,868 (median €6,225, IQR 4,064—9,721, *p* < 0.001) versus €4,528 (median €3,182, IQR 1,387–5,878) in patients with no hospitalisations.

**Table 3:** Mean annual diabetes-associated cost per patient in the study population, stratified by treatment form

	**All children** **(*n* = 5,474)**	**No technology use** **(*n* = 2,295)**	**Insulin pump** **(*n* = 1,571)**	**rtCGM** **(*n* = 116)**	**Pump & rtCGM** **(*n* = 1,492)**	**p**^†^
Diabetes-associated costs	€ 5,143 (1,570 — 6,747)	€ 2,418 (808—2,702)	€ 4,759 ^***^ (2,980—5,728)	€ 7,259 ^***^ (3,624—9,999)	€ 9,573 ^***^ (5,250—11,945)	< 0.001
Consultation costs	€ 1,049 (784—1,359)	€ 932 (459—1,292)	€ 1,137 ^***^ (902—1,372)	€ 1,030 (588—1,362)	€ 1,140 ^***^ (910—1,372)	< 0.001
Clinical costs	€ 607 (0–0)	€ 737 (0—0)	€ 473 ^***^ (0—0)	€ 1,052 (0—0)	€ 514 ^***^ (0—0)	< 0.001
Treatment costs	€ 3,179 (186—4,221)	€ 491 (0—614)	€ 2,907 ^***^ (1,600—3,516)	€ 4,137 ^***^ (876—5,885)	€ 7,525 ^***^ (3,626—9,716)	< 0.001
Diagnostic costs	€ 307 (121—297)	€ 258 (80—294)	€ 241 ^***^ (142—266)	€ 1,040 ^***^ (204—1,422)	€ 393 ^***^ (138—331)	< 0.001
Additional costs	€ 1 (0—0)	€ 1 (0—0)	€ 0 (0—0)	€ 0 (0—0)	€ 2 (0—0)	0.72

Data are presented as mean (interquartile range) costs per child, between 2019–2020. Patients were included in the stratified groups when ≥ 1 related care activity was registered. Device costs of insulin pumps and consumables like test strips, pens, and needles are not included in Dutch hospital costs. Median costs in supplementary table S3

*rtCGM* Real-Time Continuous Glucose Monitoring

^†^Difference between all technology use groups

^*^ *p* < 0.05

^**^ *p* < 0.01

^***^ 0 < 0.001, compared with no technology users

### Age and diabetes-associated costs

**Fig. 2:**
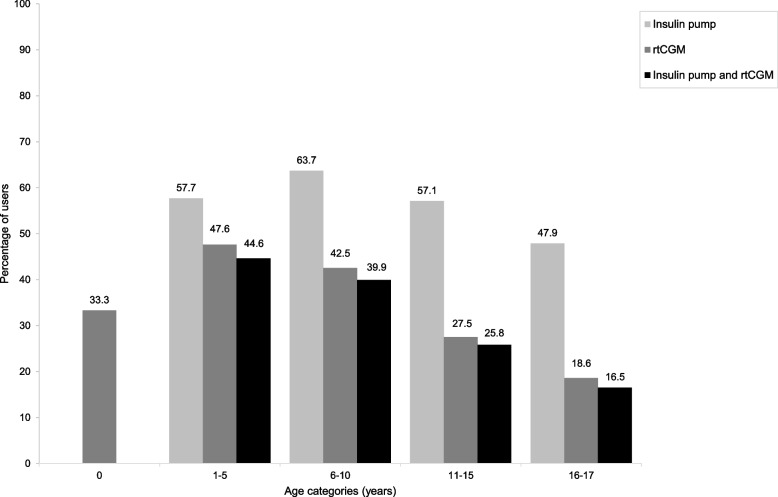
Diabetes technology utilization in different age categories

Table 4 shows mean annual diabetes-associated costs stratified by age category. Significant differences were observed in all cost subtypes except additional costs between age groups (*p* < 0.001). Mean annual costs were highest in children between 1–5 years old and lowest in children of 16–17 years old (also shown in supplementary figure S1). The group of 0 year old children (*n* = 6) had the highest percentage of treatment and diagnostic costs. The children of 1–5 years old had a relatively high contribution of clinical costs (17.8%). In all age categories, treatment costs consistently had the highest contribution to overall costs (55.1—70.8%). Figure 2 shows the utilization of diabetes technology across different ages, expressed as the percentage of users per age category. The use of technology played a role at all ages but was highest in children between 1–10 years old and lowest in adolescents. For insulin pumps, this was highest in 6–10 year olds (63.7%) and lowest in 16–17 year olds (47.9%). In case of rtCGM, this was 47.6% in 1–5 year olds and 18.6% in 16–17 year olds. In line, the treatment costs decreased with age, whereas consultation costs increased with age.

**Table 4:** Mean annual diabetes-associated cost per patient in the study population with different cost categories, stratified by age group

	**0** **(*n* = 6)**	**1–5** **(*n* = 336)**	**6–10** **(*n* = 1,119)**	**11–15** **(*n* = 2,528)**	**16–17** **(*n* = 1,485)**	**p**
Diabetes-associated costs	€ 4,239 (671—5,502)	€ 8,285 (1,885—11,530)	€ 6,564 (2,281—9,036)	€ 4,941 (1,673—6,493)	€ 3,708 (1,252—4,904)	< 0.001
Consultation costs (%)	9.6	12.4	16.7	21.6	26.7	< 0.001
Clinical costs (%)	6.1	17.8	9.7	12.1	11.0	< 0.001
Treatment costs (%)	70.8	65.0	68.9	59.9	55.1	< 0.001
Diagnostic costs (%)	13.5	4.8	4.7	6.4	7.2	< 0.001
Additional costs (%)	0.0	0.0	0.0	0.0	0.0	0.757

Data are presented as mean (interquartile range) cost per child and percentages of the mean per cost category between 2019–2020. Patients were stratified into age groups

Median costs in supplementary table S4

## Discussion

This study investigates healthcare utilization and concomitant in-hospital and outpatient costs in children with diabetes mellitus in the Netherlands. Total hospital costs for patients with diabetes were €33,002,652, and diabetes-associated costs determined most of the annual costs. Diabetes-associated costs were € 28,151,381, with corresponding mean costs of €5,143 per child. The largest share of diabetes-associated costs in children is related to the treatment of diabetes (61.8%), a cost category mainly driven by activities related to technologic devices. This study showed that indeed mean diabetes-associated costs are substantially higher for patients that use insulin pumps or rtCGM devices. During one year of follow-up, 56.0% of patients used an insulin pump, 29.4% used rtCGM, and 27.3% of patients used both treatment modalities. All-cause hospitalisation rates were lower in the majority of technology users. Across all age groups, treatment-associated costs and technology use formed the lion’s share of pediatric healthcare consumption.

Demographic characteristics of our population were in line with previous studies in children with type 1 diabetes mellitus (T1DM) in Western countries, with a slight male predominance and similar age distribution [28]. As expected, almost all children were treated by a pediatrician, which is in concordance with Dutch guidelines. Also in line with (inter) national guidelines was the screening for complications, with 20% of children who had visited an ophthalmologist [29].

Regarding overall costs, a previous Dutch study on reimbursement data showed higher mean costs of €8,326 per child compared to the current results [19]. An explanation may be the inclusion of care categories such as dental and primary care. However, mean cost per child related to secondary care was €3,119, which is lower than the current mean estimate of €5,143. Other countries have observed mean annual costs ranging between €2,712—€8,326 and $4.730—$24.093. The variation in costs in these studies may be caused by differences in the population, collection of cost data, included cost categories, national guidelines, and financial reimbursement structures. Regardless, technologic advances and increased usage of CGM in recent years can also be a reason for the increased cost estimate [28]. In several cost analyses in children, technology use was an important contributor to costs [9, 10, 12, 13, 15, 16]. In line with these studies, we observed a significant increase in costs when stratified by treatment regimen.

The high percentage of children using insulin pumps was similar to German and Austrian children and a privately insured cohort in the USA [9, 30]. The Swedish national quality register showed that 64.7% of children used insulin pumps, yet the global SWEET registry observed lower usage of 41.8% across 19 countries [31, 32]. Surprisingly, our results suggest the use of insulin pumps to be comparable to other countries, yet the use of CGM devices was lower. In German and Austrian children in 2017 overall CGM use was 38%, and in participating SWEET centers 44.6% (95% CI 2.3 – 52.4%) was observed [30, 32]. The percentage of CGM users in Sweden was as high as 93% in 2018 [31]. In concordance with previous studies, the use of CGM was the lowest in adolescents (16.5% of 16 to 17-year-olds) and emphasizes the challenges in this age group [28, 33]. This may also be the reason for the low observed diabetes-associated costs in this group. We can only speculate on the reason for the decrease in these treatment costs because the cost data were only specified on a category level. It is known that barriers related to self-image, costs, inconsistent use, and differences associated with ethnicity and socio-economic status all influence technology utilization among adolescents and young adults [33]. Additionally, it is possible that newly diagnosed patients are treated with more technology or better technology. Moreover, lower uptake percentages in the overall study population may, to some degree, be caused by the exclusion of intermittently scanned CGM (isCGM) in the current results. IsCGM is not systematically recorded in the Dutch reimbursement system, causing under registration of isCGM and total CGM use. Financial barriers may also have played a role in the relatively low number of CGM users because reimbursement of rtCGM devices was limited by local hospital budgets until the end of 2020. Device costs of insulin pumps and isCGM are financed under a different structure called resource care, and this can also explain lower hospital costs in insulin pump users. These outcomes illustrate the complexity of reimbursement of diabetes technology and the influence of policymaking, financial structures, and insurance companies on healthcare utilization. In fact, coverage differences are known to cause heterogeneity in technology use in children across Europe [34]. Local reimbursement was indeed found to influence pediatric endocrinologists in recommendation of diabetes technology to their patients [35]. As of 2021, national policy changes have fortunately removed rtCGM devices from hospital budgets. By rendering rtCGM devices ubiquitously available, this will hopefully improve accessibility for all Dutch patients, but may also increase future costs. Evolving technologic opportunities like CGM facilitate better (self-)management of diabetes, improve glycemic control and result in better psychosocial outcomes [8, 33]. Increasing costs associated with modern technology use may therefore be compensated by improvements in patients’ daily lives. In line, the current results show that all-cause hospitalisation rates decreased in most technology users. It is plausible that CGM or combined pump and CGM use will also result in cost saving in the long run by reducing complications and mortality, but this remains to be assessed in future cost-effectiveness studies in children.

Our study also underlines shortcomings in the national registration process. Benchmarking on a national and international level improves quality of care and long-term outcomes, but this is impossible without a clear overview of provided care [32]. Efforts should be made to further improve registration to ensure adequate monitoring of consumption, thus enabling evaluation of newer therapy forms. Policy makers should be aware of the importance of adequate registration and of barriers for patients caused by complexity of reimbursement policies.

This study shows real-world data on healthcare resource utilization in combination with costs in Dutch children with diabetes mellitus, representing different ages, regions and socio-economic backgrounds by using data from the majority of all Dutch pediatric patients. The large sample on a national level makes it possible to identify generalizable associations. Moreover, this study was able to report the use of healthcare resources and diabetes technology in a population with insurance coverage for all children. In our results, an acting cost methodology was used to enable benchmarking between hospitals and therefore express factual estimations of costs without the influence of price negotiations.

There also were limitations to this study. As current data is used primarily for administrative and benchmark purposes, only selective data was available, and registration errors could not be omitted. As Dutch reimbursement codes are not specified by diabetes type, no distinction could be made between T1DM and type 2 (T2DM) diabetes. However, general practice estimates show it is reasonable to assume that almost all of the children in the study population are diagnosed with T1DM because the prevalence of T2DM in Dutch children is relatively low [4]. Furthermore, only data from affiliated hospitals were used, and the resulting absence of independent treatment clinics may have introduced selection bias. Moreover, data from only one year of follow-up was available, and no information on clinical parameters or reason for hospitalisation was present. It is known that glycemic control, duration of diabetes, complications, and insulin use may influence costs and healthcare use [9, 10, 14, 16]. The COVID-19 pandemic may have had an impact on clinical care and, therefore on our results; especially the number of admissions and consultations in 2020 may have been affected. In several studies, an increase in severe DKA was observed in new-onset diabetes during the COVID pandemic [36, 37]. Regardless, telephonic consultations were also included, and the number of hospitalised children was still relatively low. Despite the mentioned limitations, we believe that the results provide a realistic insight into current pediatric diabetes practice in a Western country with insurance for all children.

The current findings illustrate that attempts should be made to lower the costs of diabetes technology, at least until better treatment options for children with T1DM become available. Efficient use of technology can also help reduce overall expenditure through its application to reduce hospitalisations and related costs. Despite high treatment and clinical costs in young children, optimal and device-intensive treatment of this patient group remains important to prevent future morbidity and mortality [6, 7]. Meanwhile, other treatment options, such as a patient-centered approach and multidisciplinary care teams, may help to strengthen pediatric diabetes practice further. 

## Conclusions

To conclude, this study provides an overview of current healthcare utilization and concomitant costs in children with diabetes from a nationwide perspective. Regardless of age, hospital costs are mainly driven by the treatment of diabetes, and technology use was of significant influence. Compared to neighbouring countries, the use of insulin pumps was comparable, but the use of rtCGM was relatively low. Mean costs were significantly higher among most children using diabetes technology, but hospitalisation rates and costs decreased. Increased costs of modern technology use may likely be compensated with improved long-term outcomes and quality of life, yet also highlight the importance of adequate registration and cost-effectiveness studies.

## Supplementary Information

**Additional file 1:**

## Data Availability

The dataset generated and analysed during the current study is not publicly available due to the sensitive nature (license restrictions, commercial and privacy regulations) of the data. Data are however available from the corresponding author upon reasonable request and with permission of a third party (LOGEX).

## Abbreviations

DBC: Diagnosis Treatment Combinations
HIS: Hospital information systems (HIS)
SES: Socio-economic status (SES)
CPI: Consumer Price Index
HCRU: Healthcare resource utilization
CGM: Continuous Glucose Monitoring
rtCGM: Real-time Continuous Glucose Monitoring
isCGM: Intermittently scanned CGM
