# Supplementary material for: Healthcare expenditure and technology use in pediatric diabetes care
Source: BMC Endocr Disord. 2023 Apr 7;23:72. doi: 10.1186/s12902-023-01316-3 (PMC10080182; doi:10.1186/s12902-023-01316-3)
Supplement: Supplementary file 1 — Additional file 1: [file 12902_2023_1316_MOESM1_ESM.docx]

# **Supplemental File**

**Table S1.** Included Dutch specialty codes and corresponding diabetes diagnosis codes

| **Hospital based specialty code** | **Diabetes diagnosis code** |
| --- | --- |
| Pediatrics [0316] | [7113 or 7114] |
| Internal medicine [0313] | [221, 222 or 223] |
| Surgery [0303] | Diabetic foot diagnosis code [432] |
| Orthopedics [0305] | Diabetic foot diagnosis code [2065] |
| Ophthalmology [0301] | Diagnosis codes for diabetic retinopathy and maculopathy [754, 751, 755, 759, 757, 705] |

**Table S2.** Included Dutch healthcare activity codes related to technology use

| **Insulin pumps** | **rtCGM use** |
| --- | --- |
| 35520 | 39575 |
| 35523 | 39583 |
| 35524 | 39628 |
| 35525 | 39641 |
| 39582 |  |
| 190309 |  |

**Table S3.** Median annual diabetes-associated cost per patient in the study population, stratified by treatment form

|  | **All children  (n=5,474)** | **No technology use  (n=2,295)** | **Insulin pump  (n=1,571)** | **rtCGM (n=116)** | **Pump & rtCGM (n=1,492)** | **p**† |
| --- | --- | --- | --- | --- | --- | --- |
| Diabetes-associated costs | € 3,711  (1,570 – 6,747) | € 1,506  (808 - 2,702) | € 4,009 ***  (2,980 - 5,728) | € 6,474 ***  (3,624 - 9,999) | € 7,911 ***  (5,250 - 11,945) | <0.001 |
| Consultation costs | € 1,060  (784-1,359) | € 980  (459 - 1,292) | € 1,163 ***  (902 - 1,372) | € 1,007  (588 - 1,362) | € 1,114 ***  (910 - 1,372) | <0.001 |
| Clinical costs | € 0  (0-0) | € 0  (0 - 0) | € 0 ***  (0 - 0) | € 0  (0 - 0) | € 0 ***  (0 - 0) | <0.001 |
| Treatment costs | € 1,711  (186-4,221) | € 124  (0 - 614) | € 2,351 ***  (1,600 - 3,516) | € 2,805 ***  (876 - 5,885) | € 5,821 ***  (3,626 - 9,716) | <0.001 |
| Diagnostic costs | € 194  (121-297) | € 180  (80 - 294) | € 197 ***  (142 - 266) | € 868 ***  (204 - 1,422) | € 202 ***  (138 - 331) | <0.001 |
| Additional costs | € 0  (0 - 0) | € 0  (0 - 0) | € 0  (0 - 0) | € 0  (0 - 0) | € 0  (0 - 0) | 0.72 |
| *Data are presented as median (interquartile range) costs per child, between 2019-2020. Patients were included in the stratified groups when ≥1 related care activity was registered. Device costs of insulin pumps and consumables like test strips, pens, needles are not included in Dutch hospital costs.* | | | | | | |
| *rtCGM = Real-Time Continuous Glucose Monitoring* | | | | | | |
| †*Difference between all technology use groups* | | | | | | |
| ** p<0.05, ** p<0.01, *** 0<0.001, compared with no technology users* | | | | | | |

**Table S4.** Median annual diabetes-associated cost per patient in the study population with different cost categories, stratified by age group

|  | **0 (n=6)** | **1-5 (n=336)** | **6-10 (n=1,119)** | **11-15 (n=2,528)** | **16-17 (n=1,485)** | **p** |
| --- | --- | --- | --- | --- | --- | --- |
| Diabetes-associated costs | € 2,136  (671 - 5,502) | € 5,929  (1,885 - 11,530) | € 4,921  (2,281 - 9,036) | € 3,727  (1,673 - 6,493) | € 2,715  (1,252 - 4,904) | <0.001 |
| *Data are presented as median (interquartile range) costs per child between 2019-2020. Patients were stratified in age groups.* | | | | | | |

**Figure S1.** Mean total and diabetes-associated costs per patient in the study population with different cost categories, stratified by age group

DM = diabetes-associated
